# Multivariate Characterization of Temperature Fluctuations in a Historical Building Using Energy-Efficient IoT Wireless Sensors

**DOI:** 10.3390/s21237795

**Published:** 2021-11-23

**Authors:** Manuel Zarzo, Angel Perles, Ricardo Mercado, Fernando-Juan García-Diego

**Affiliations:** 1 Department of Applied Statistics, Operations Research and Quality, Universitat Politècnica de València, Camino de Vera s/n, 46022 Valencia, Spain; mazarcas@eio.upv.es; 2 ITACA Institute, Universitat Politècnica de València, 46022 Valencia, Spain; rmercado@itaca.upv.es; 3 Department of Applied Physics (U.D. Industrial Engineering), Universitat Politècnica de València, 46022 Valencia, Spain; fjgarcid@upv.es

**Keywords:** microclimate monitoring, preventive conservation, heritage buildings, Internet of Things

## Abstract

Adequate thermic conditions are required for the preventive conservation of artworks, but such optimum conditions cannot always be achieved in historical buildings such as ancient churches. In those cases, it is of interest to assess the potential risk of punctual changes in indoor environments that can be harmful to artworks. These conditions can be assessed by means of a microclimate monitoring system comprised of a set of energy-efficient wireless sensors connected to the cloud using IoT techniques. This approach was followed at the baroque church of Saint Thomas and Saint Philip Neri in Valencia (Spain). A set of 26 wireless nodes was installed, which recorded values of temperature and relative humidity every hour for a period of 7 months. Small differences of temperature were obtained among sensors, so that an efficient methodology based on principal component analysis (PCA) was applied for the characterization of similarities and dissimilarities between sensors. Daily ranges of temperatures were studied as well as mean trajectories, differences between days of the week, and changes in the correlation structure of daily median values over time. Results provide a framework for an efficient characterization of temperatures in heritage buildings based on a network of wireless sensors. Such a framework is useful to assess the potential risk of temperature fluctuations on the preventive conservation of historical buildings and artworks.

## 1. Introduction

In 2018, the “travel and tourism” sector contributed directly to the European Union’s gross domestic product (GDP) at a rate of 3.9% and accounted for 5.1% of the total workforce, representing about 11.9 million jobs. If other economic sectors related to “travel and tourism” are taken into account, the contribution of the tourism sector is even higher (10.3% of GDP and 11.7% of total employment, namely 27.3 million workers) [1].

Undoubtedly, cultural heritage (CH) is an important tourist attraction in Europe. To achieve the conservation of CH, the European Union is investing in important research projects. The preventive conservation of artworks by keeping an adequate microclimate allows an extension of their useful life as objects that can be contemplated by visitors and, secondly, delays expensive future restorations in time [2,3].

In order to reach this objective, it is necessary to monitor the microclimatic conditions, which have been widely discussed [4,5,6] and set out in standards [3,7,8,9,10,11,12].

The current European standard EN 15757:2010 [7] specifies relative humidity (RH) requirements to limit climate-induced mechanical damage in organic hygroscopic materials. This standard provides a new philosophy compared to former guidelines such as the Italian standard UNI 10829:1999. In the latter, fixed values of boundary limits were proposed for the conservation of artworks. Most old churches in Spain lack centralized climatization systems, so that it is not possible to maintain the inner temperature between certain limits in order to optimize the conservation conditions for artworks. This approach requires powerful air conditioning systems to fulfil the requirements, which implies high energy consumption and precludes the application of UNI 10829:1999 in many CH scenarios, such as big ancient churches or semi-confined archaeological sites [4,13,14]. By contrast, in EN 15757:2010, it is emphasized that if a piece of art has reached our days under changing microclimate conditions, due to marked seasonal and daily variations, such unsteady conditions are not so detrimental if they are maintained at the same rate. As a consequence, this approach allows a reduction in both carbon footprint and energy use [11,15]. This philosophy is the basis of the concept of green museums [16].

The European Standard EN 15758:2010 [3] explains the role played by air temperature in the conservation of objects, as well as the temperature of their surface. According to this standard:

- The materials of which the works are made can absorb or give off heat. Objects expand or contract as a function of temperature variations, becoming rigid and brittle if the temperature falls below the glass transition temperature.
- The speed of some important chemical reactions, such as the degradation of cellulose (e.g., paper, textiles, wood), increases with increasing temperature.
- Temperature influences the activity of fungi and insects responsible for the biological deterioration of organic materials.
- It can also affect certain minerals and crystallization phenomena in masonry factories.
- As an important indirect effect, a rise in temperature causes a decrease in relative humidity (also cited in EN 15757:2010), which implies the drying out of hygroscopic materials such as wood, paper, or leather. This loss of moisture can cause shrinkage and embrittlement of materials.
- When objects are exposed to direct sunlight, lamps, or heating radiators, the consequent rise in temperature causes drying, even if the relative humidity of the surrounding air remains constant.
- Water vapor can condense on cold surfaces if their temperature falls below the dew point, which is a problem because the presence of liquid water can be harmful for the preservation of many materials.

To date, peer reviewed literature has given great attention to hygrothermal microclimate in CH. The preventive conservation in museum buildings has been reviewed by [17]. Other studies have also discussed the appropriate environments in museums [18,19]. Different authors, such as Camuffo [20,21], Lucchi [22,23], Beccherini [24], Schito [25,26], Silva [27,28,29], and others [30], have studied temperature variations in temperate climates of the Mediterranean region. 

The effect of temperature variability is one important issue that is less considered in EN 15757:2010 [31]. Moreover, given that churches represent a small percentage of energy expenditure, it turns out that the monitoring of T in churches has not received much attention as a fundamental parameter to be considered by the governments. Other authors have studied thermal indoor microclimate conditions in CH, referring to both the preservation of artworks and the comfort of visitors or workers at these buildings [32,33].

Temperature data can be collected in many different ways, e.g., using wired sensors [34,35] and dataloggers [36,37,38]. A recent study has proposed a 3D thermal technology for heritage buildings [39]. Because of thick stone walls and long distances, wireless communications within heritage sites are often complicated and some authors have abandoned them after testing [40], though wireless systems are still being investigated to overcome the drawbacks [41,42]. Other researchers have studied a methodology for assessing the quality of wireless communications and validating the network used, both of which are essential for guaranteeing accurate long-term monitoring in heritage scenarios [43].

Multivariate statistical methods such as principal component analysis (PCA) [35] and cluster analysis (CA) [6] have demonstrated their usefulness for the characterization of microclimatic air conditions or in discussing the number of sensors to be used [44]. Both statistical tools have demonstrated a challenging capability for classifying time series with very similar patterns and for optimizing the number of sensors required to monitor the indoor air conditions in museums. Time-frequency analysis is another well-researched domain for the classification of sensor data [45,46,47].

Autonomous dataloggers are relatively cheap nowadays, but they require the manual download of data, which can be time consuming. An alternative approach is to use sensors with wireless technology compatible with IoT. This technology was applied in the baroque church of Saint Thomas and Saint Philip Neri in Valencia (Spain) as a case study [48].

This church was built between 1727 and 1736 and contains an important collection of artworks, including paintings by renowned Spanish artists such as Juan de Juanes (1507–1579), Jerónimo Jacinto de Espinosa (1600–1667), José Vergara (1726–1799), and Vicente López (1772–1850). Hence, there is an interest in evaluating the microclimatic conditions, aimed at discussing the preventive conservation of the valuable artworks conserved in this church.

Most old churches in Spain lack centralized climatization systems, so it is not possible to maintain the inner temperature between certain limits in order to optimize the conservation conditions for artworks.

The present work has analyzed temperature values recorded by a monitoring system based on IoT, previously developed by our team [48], to have a fully functional demonstrator to test these techniques and to also address the problems identified by other authors in similar situations [40,49,50,51]. With this monitoring experiment, comprised of 26 wireless sensor nodes, we intended to demonstrate the usefulness of this technology by collecting the temperatures at different points and heights of the church. The main objective of this work is to discuss the advantages of this technology using PCA for characterizing the different temperatures recorded by sensors according to their position, especially height and distance from the principal entry, which is the main source of airflow exchange. Results provide useful information to discuss the appropriate number of sensors used for monitoring, their optimal location, and the importance of sensor calibration. Using a set of 26 nodes is obviously excessive for the monitoring of air conditions that are rather stable as in this case, because the church has a low ventilation rate and the central nave is not heated. Nonetheless, a deep understanding of the factors explaining the temperature variability is necessary with a preliminary study such as this research work, in order to establish the optimum number of sensors.

## 2. Materials and Methods

As described in the introduction, wireless environmental monitoring techniques show promise in dealing with the usual characteristics of historic buildings, which are often large and thick walled. In order to explore the potential of these technologies in the field of cultural heritage conservation, a wireless monitoring system was developed to assess indoor air conditions at the baroque church of Saint Thomas and Saint Philip Neri in Valencia [48].

### 2.1. Description of the Microclimate Monitoring System

In the design of the monitoring system, it was decided that it should consist of ultra-low power autonomous wireless sensors using transmission techniques capable of coping with the particularities of historic buildings and, at the same time, that the batteries should last for years without the need for maintenance. For data collection, a gateway should be developed capable of receiving sensor transmissions, aggregating them, pre-processing them, and transmitting them to the cloud via an Internet connection. Finally, an infrastructure should be provided to store and process environmental data in real time, which should always be available and accessible by the user. Thus, it was decided that the best option was to deploy a cloud computing system accessible to users through the Internet via a web browser. This approach is in line with the concept of the Internet of Things (IoT) and is rapidly being incorporated into smart building solutions [52].

**Figure 1:**
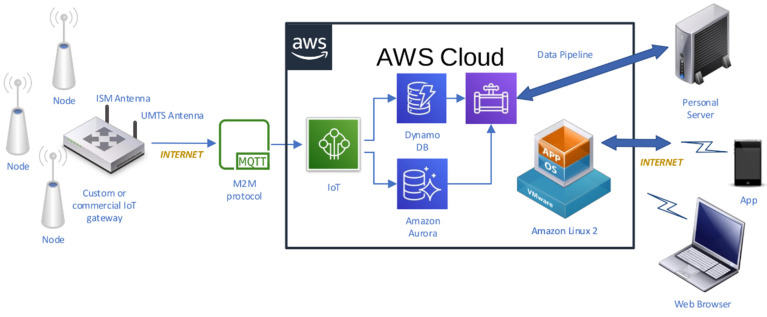
Diagram of the wireless microclimate monitoring system.

As a result of these requirements, a system was designed and deployed, the diagram of which is shown in Figure 1. It is comprised of ultra-low power wireless sensor nodes, a sink gateway in charge of collecting data transmissions, and cloud infrastructure. 

**Figure 2:**
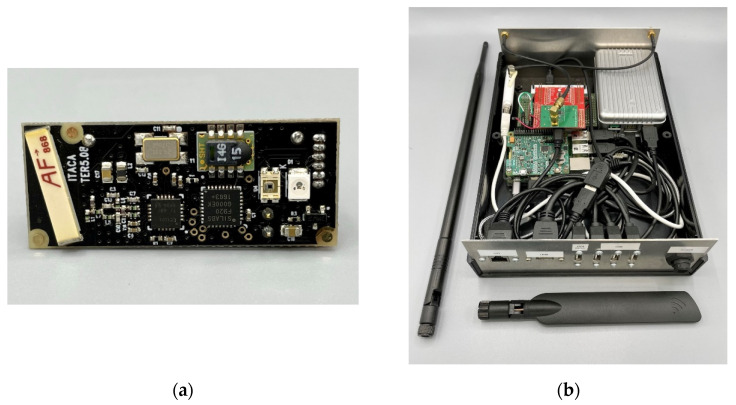
Key components of the electronics associated to the monitoring system: (**a**) wireless sensor node with a size of 4 cm × 1.5 cm × 1.5 cm, (**b**) custom gateway designed specifically for the monitoring needs in cultural heritage sites.

The wireless sensor nodes, depicted in Figure 2a, are built around an ultra-low power C8051F920 microcontroller (Silicon Laboratories Inc., San José, CA, USA), a SHT15 temperature and humidity sensor (Sensirion, Staefa ZH, Switzerland) with a typical accuracy of 0.3 °C, a CC1101 radio modem (Texas Instruments, DA, USA), and a high-density 3.6 V, 1 Ah Lithium-thionyl battery. Data are transferred wirelessly to the gateway using the 868 MHz European unlicensed industrial, scientific, medical (ISM) band, Gaussian frequency-shift keying (GFSK) for the modulation, and a fixed transmission frequency for all the sensor nodes. This sensor node is an adaptation of a previous one devoted to the detection of xylophagous [53] that copes adequately with the requirements of lifespan and long distances and tick walls of historical buildings.

The gateway, shown in Figure 2b, was built to be as flexible as possible in order to experiment with different approaches, so it was decided to implement it around a Raspberry Pi 3 board (Broadcom Inc., San Jose, CA, USA) and the Linux operating systems. Suitable hardware was added to this base system in order to support the functionality: a CC1101 radio module and an STM32L04 microcontroller (STMicroelectronics N.V., Geneva, Switzerland) to receive the transmissions of the sensor nodes, and a 3G USB dongle to provide mobile connectivity to Internet, and, considering that the gateway is connected to the mains power, a rechargeable lithium-ion battery to provide energy to the gateway during power outages. The main task of the gateway is to collect wireless transmissions of the sensor nodes, store them temporarily in a local database, and transmit them to Internet when connectivity is available. The data transfer is implemented using the MQTT [54] client server publish/subscribe messaging transport protocol.

For the implementation of the cloud infrastructure, it was decided to choose the offering from Amazon Web Services (AWS). The MQTT messages are processed by the AWS IoT cloud service in order to split the message in sensed magnitudes such as temperature, humidity, or light level (humidity and light not used in this work), as well as in communication-related parameters (e.g., received signal strength indicator, battery level, and message counter). These two types of data flows are stored in a NoSQL AWS DynamoDB database and in an SQL AWS AuroraDB, respectively. In order to allow data access through web browsers, a Linux virtual machine was deployed in the AWS EC2 service which runs a Redash [55] data visualization dashboard. For statistical analysis, all data collected along the monitored period could be downloaded locally using AWS Data Pipeline service. For the ambient sampling strategy of the monitoring system, the time between two consecutive measurements (and transmissions) was set as a random variable following an exponential statistical distribution with a mean of one hour; the initial purpose of this transmission pattern was to decrease the probability of data transmission collisions and to provide statistically independent sample points. As described below, this strategy became problematic for the data treatment, so it has been discarded in subsequent designs, following fixed sampling patterns based on standards such as UNI 10829:1999 [56], EN 15757:2010 [7] and ASHRAE 2011 [57]. As a reference, [58] reviews the standards and procedures suitable for creating a monitoring system dedicated to cultural heritage. In that work, a wired solution with 3G connectivity is proposed to cover specific needs that this particular IoT solution cannot cover, e.g., sampling rate, and equipment redundancy.

### 2.2. Sensor Calibration

When a set of thermic sensors is implemented for monitoring a confined environment, small differences are expected among the sensors. As undertaken by other authors [59,60], a bias correction was performed prior to their installation by considering the mean value of all sensors as the true temperature according to a calibration experiment carried out. Firstly, 26 sensor nodes were placed together inside a climate chamber of 23 m^3^ driven by a ceiling air cooler (Küba Comfort DP model DPB034) at a controlled temperature, increasing from 26 up to 30 °C for three hours. Next, the temperature inside the chamber was reduced to 16 °C for less than one hour. This calibration experiment lasted for 320 min, and the number of measurements recorded by each sensor fluctuated between 491 and 550, which implies one value collected every 35–41 s.

Once the experiment finished, the mean temperature recorded by each sensor was computed for both the hot and cold stages. Next, the correlation between both parameters was checked in order to discuss if the bias resulting from the calibration at the cool stage was similar to that obtained at the hot stage. This bias was calculated for each sensor by subtracting the mean temperature recorded by that node with respect to the grand mean collected by all nodes together. Finally, all temperatures collected during the microclimate monitoring experiment by a given node were corrected by subtracting the corresponding bias.

**Figure 3:**
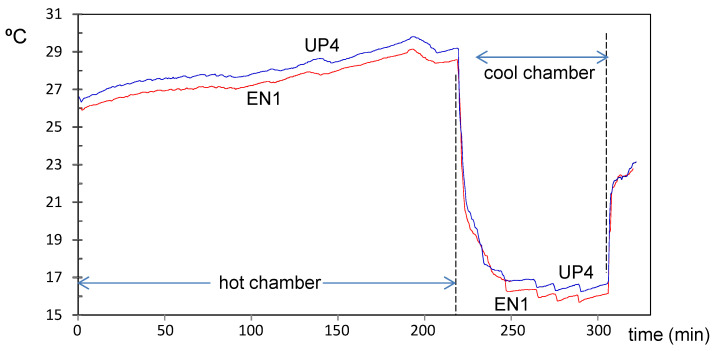
Trajectories of temperature during the calibration experiment, corresponding to nodes EN1 (in red) and UP4 (in blue). A parallel pattern is observed both for the hot and cool stages.

For each node, the mean temperature (T_mean_) during the calibration in the hot stage was computed by averaging the first 320 values. Next, the minimum temperature was obtained for the cool stage. It turned out that both parameters are positively correlated (*r* = 0.743, *p* < 0.0001), which suggests that trajectories from both calibration stages are approximately parallel for the set of sensors. In the hot stage, the node marked as UP4 (node labels described in the next section) was the node with highest mean (T_mean_ = 28.09 °C), while the lowest mean corresponded to EN1, T_mean_ = 27.48 °C. Figure 3 shows the time series of temperature in the calibration experiment for both nodes. Their trajectories are parallel during the whole experiment, which implies that the sensor bias can be assumed as equal for the hot and cool stages (i.e., the bias does not depend on temperature).

The difference between T_mean_ of UP4 and EN1 is 0.61 °C, which is consistent with the accuracy of the Sensirion SHT15 (±0.3 °C) in the range of temperatures used for the calibration. In this design, it was decided to select the most accurate model of the SHT1x series, in contrast to previous projects [53] where Sensirion SHT10 was utilized to optimize the cost of the solution. Currently, Sensirion SHT1x series are currently deprecated, based on our experience we would recommend using Sensirion SHT3x series sensors for the new designs.

By comparing T_mean_ values of each node, derived from the calibration experiment, it was found that the T_mean_ of RE4 was the one closer to the grand mean calculated using all nodes. Hence, a null bias was considered for RE4.

As the calibration experiment at the hot stage lasted longer than the one at the cool stage, the estimated bias is more accurate. Thus, it was decided to compute the bias of each sensor by subtracting the mean temperature recorded during the hot stage (T_hot_) minus the T_hot_ of the reference node RE4. It was checked that the resulting temperature biases (Table 1) follow, approximately, a normal distribution with mean equal to zero and standard deviation *s* = 0.16. Given that the present research intends to discuss small differences of temperature, it is necessary to achieve the maximum accuracy in all measurements. Thus, the original values were corrected with the bias corresponding to each node, by subtracting every recorded value minus the bias indicated in Table 1.

The calibration procedure used in this work might be arguable because a reference sensor calibrated by the manufacturer should be required for an accurate estimation of the real bias [61]. Nevertheless, the present work intends to characterize the relative differences of temperature inside the building, and hence knowing the exact real bias of each sensor is of no interest in this case. Other authors [59,60] have found that this approach is adequate for making comparisons between sensors.

In [58], different thermal insulation materials were tested, and a reliable calibration was needed for this purpose. To achieve this goal, it is necessary to know the true value of temperature with precision. So, precalibrated industrial sensors with high accuracy were selected. In the present work, this is not necessary because PCA deals with differences and not with the true values.

### 2.3. Installation of Wireless Sensor Nodes

A total of 26 sensors were installed in the church. This number is a balance between budget availability and the size of the building to be monitored. Another work [58] used an infrared thermography method for deciding the best position of sensors. This method helps to map the superficial thermal conditions of the walls; therefore, it is a qualitative way to verify the absence of thermal anomalies in the original wall. In the present work, this approach is not suitable because we are studying air temperature differences. 

**Figure 4:**
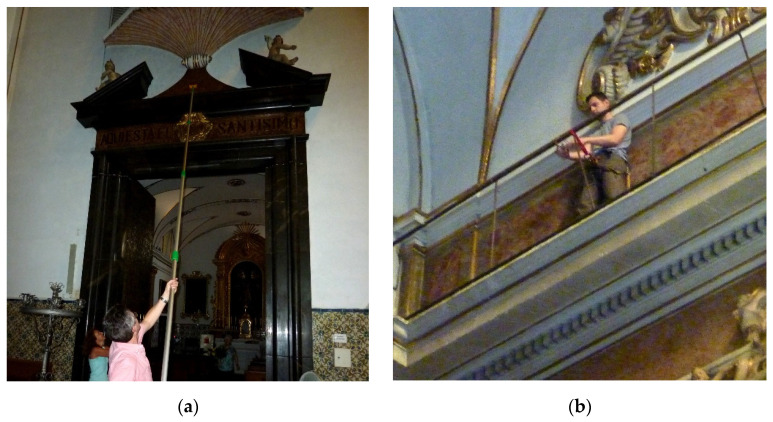
(**a**) Installation of sensor nodes at heights up to 9 m. (**b**) Installation of the sensor nodes clamped at above 12 m to the security banister of the cornice on top of all pilaster capitals around the entire perimeter of the central nave.

As indicated in the description of the monitoring system, the aim was also to make the deployment as quick and simple as possible in order to minimize interference with the tempo services and, of course, without affecting the state of conservation of artworks and buildings. In this sense, the set-up of the gateway consisted of simply powering it on, and the installation of the sensors consisted of dropping them at the point of interest manually or by means of a pole (Figure 4a). However, for those located at higher elevations, specialized personnel were required to clamp them to the security banister of the cornice on top of all pilaster capitals around the entire perimeter of the central nave (Figure 4b). One morning was spent on the installation of the lower nodes and another morning deploying the upper nodes.

**Figure 5:**
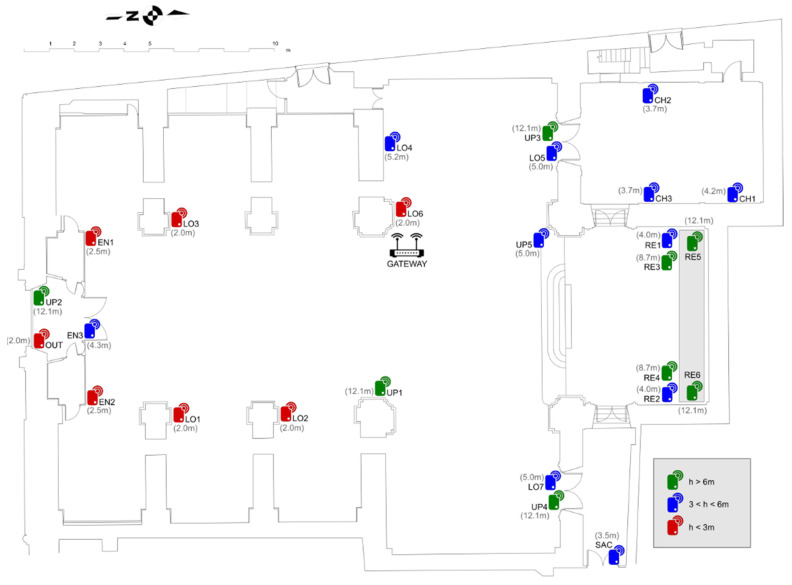
Position of the 26 nodes installed at the church of Saint Thomas and Saint Philip Neri (Valencia). Color codes according to their height (h): red (h < 3 m), blue (3 < h < 6 m), green (h > 6 m). The position of the sink gateway is indicated as SG. The retable is depicted in grey.

Several criteria were considered to decide the position of nodes given the quantity of available nodes. Figure 5 shows positions and heights of the sensor nodes. For example, six of them, coded as UP1 to UP4 and R5, R6, were located at heights higher than 12.1 m in order to assess the upper environment close to the ceiling vaults; three nodes were placed at the Communion Chapel (CH); six at the retable decorating the presbytery (RE); seven were spread out at different low points (LO) of the central nave (at a height between 2 and 3 m); and three were conveniently positioned to evaluate the indoor air conditions near the main entrance (EN). Another node was located at the corridor leading to the sacristy (SAC) and the last one was positioned outside the church (OUT). Considering that the outer doors of the temple are open at least six hours per day, an additional node was installed inside the narthex (EN) in order to assess the environment here, which can be regarded as intermediate between air conditions at the central nave and outside the building. Apart from the Communion Chapel, narthex, and the corridor, which can be considered as somewhat independent chambers, the target was to characterize the indoor temperature according to height and distance from the principal entry, which is the main source of airflow exchange. Trying to prevent nodes from being manipulated unintentionally, they were properly positioned to minimize their visibility and to not be easily reached by people. For this reason, none of them were put very close to the floor level.

Table 2 indicates the height of each node. Those located at the Communion Chapel, coded as CH (chapel), are the following:

- **Node CH1**: Placed on a window ledge, at about 7 m from the chapel door. This window is east oriented and, except on cloudy days, it is heated by direct sunshine radiation incident on the glass during a certain time frame that varies throughout the year. The window remained open during part of the period under study, so that collected values were influenced by the entrance of outer air, as discussed below.
- **Node CH2**: Dropped on the top of a painting framework, at 5 m from the door and 7 m away from CH1.
- **Node CH3**: Dropped on the top of a painting framework in front of CH2 (at 6.9 m away), at 5 m from the chapel entrance.

The nodes mostly devoted for monitoring the top environment, coded as UP (upper position) are:

- **Node UP1**: It was fastened with a clamp to the security banister located 1 m above the cornice that decorates all pilaster capitals around the entire perimeter inside the central nave. This cornice has a width of about 0.5 m and allows access to some upper windows. The closest node was LO2, located 10 m away.
- **Node UP2**: Installed at the banister of the cornice, above the main entrance of the church. The closest node was EN3, which remained about 7 m below.
- **Node UP3**: At the cornice banister, at about 7 m above LO5, both located over the access door to the Communion Chapel.
- **Node UP4**: At the cornice banister in a symmetrical position with respect to UP3 and at the same height, above node LO7. Both were placed over the door that opens to a corridor leading to the sacristy.
- **Node UP5**: Dropped on the wooden sounding board covering the pulpit. It remains at 4.5 m away from LO5, which was the closest node.

The space around the high altar is usually called the chancel or presbytery. In this church, the chancel floor has an elevation of 1 m with respect to the nave floor. The wooden altarpiece behind the high altar was manufactured in 1941 and has 3 alcoves or niches with wooden statues of saints. Given the considerable size of this artwork, it was decided to install 6 nodes here, coded as RE (retable):

- **Node RE1**: Located at the left side of the retable. It was dropped on the base of the niche holding a statue of Saint Philip Neri.
- **Node RE2**: Right side of the retable, symmetrically placed with respect to RE1, on the base of the niche with a statue of Saint Vincent Ferrer.
- **Node RE3**: Left side of the retable, at 4.6 m above RE1 and 4.2 m below RE5, on the capital crowning a wooden column, which is a flat ledge that protrudes about 20 cm.
- **Node RE4**: Right side, on the symmetric capital with respect to RE3, at 8 m away.
- **Node RE5**: Placed in the nook of a volute decorating the altarpiece top, at 4.6 m above RE3. The volute was accessed by walking through the cornice decorating the entire upper perimeter of the central nave.
- **Node RE6**: On a symmetric position with respect to RE5, 8.0 m away, at 4.6 m above RE4, also in the nook of a volute decorating the altarpiece top.

Seven nodes were located at the central nave near the floor level, coded as LO (lower) are:

- **Node LO1**: Dropped on the roof of a wooden confessional, it remained at 9 m away from the main entrance. The closest node was EN2, at a distance of 6.5 m.
- **Node LO2**: On the top of a confessional at 7.4 m away from LO1, which was the nearest node.
- **Node LO3**: Located on the roof of a wooden confessional, at 9.0 m away from the main entrance. From 8 December 2017 until 2 February 2018, the nativity scene was installed next to this confessional.
- **Node LO4**: Placed at 5.2 m away from LO6, on the back side of a big picture (8.26 m × 3.86 m) from José Vergara, oil-painted on canvas dated about 1735, depicting Saint Philip Neri with pope Gregory XIII. The frame is separated about 20 cm from the wall by means of a metal structure in order to allow ventilation and improve the preservation conditions. This node was fastened to the bottom edge of the frame, facing the wall, 20 cm away.
- **Node LO5**: It was dropped on the marble lintel above the door leading to the Communion Chapel, at 7.1 m below UP3.
- **Node LO6**: Placed at the top of another wooden confessional.
- **Node LO7**: Symmetrically positioned with respect to LO5, above the door opening to a corridor that leads to the sacristy, at 7.1 m below UP4.

Some nodes were placed close to the main door, coded as EN (entrance):

- **Node EN1**: Dropped on the roof of the narthex, at 4.4 m away from EN3. This narthex is a wooden structure acting as foyer or vestibule at the church entrance.
- **Node EN2**: In a symmetrical position with respect to EN1, also on the narthex roof.
- **Node EN3**: When entering the church from outside, there is a front door that is part of the narthex, which remains closed most of the time. This node was installed on the top of such door frame, facing the main nave, at about 7.8 m below UP2.

In addition, the remaining two nodes are the following:

- **Node OUT**: Inside the narthex, dropped on the top of a frame. Although it was denoted as OUT, it, in fact, provided information about the transition environment from outside and inside the temple because the main entrance is open at least six hours every day. Unfortunately, this node disappeared on 1 September 2017 at 11:00 a.m., so temperatures are only available for the first 32 days.
- **Node SAC**: Placed at the corridor leading to the sacristy and other rooms, hanging on a nail at the top of a door frame. It remained 4 m away from the door that opens to this corridor, which is a chamber quite isolated form the central nave. Hence, it was of interest to monitor air conditions here.

The criteria for the placement of nodes were initially intended to cover the air inlet areas in the lower part, upper areas, and the entire wooden altarpiece at the high altar.

### 2.4. Data Pretreatment

The experiment of microclimate monitoring lasted for 7 months (212 days), from 1 August 2017 until 28 February 2018. The system was programmed so that each wireless node should send the recorded value to the gateway periodically.

As commented in the description of the monitoring system, the time between two consecutive measurements was set as a random variable following an exponential statistical distribution with a mean of one hour. As a result, the total number of values recorded by each sensor was not the same, which becomes problematic when trying to structure the dataset as a matrix of observations (instants of time) by variables (sensors). In order to avoid this drawback, linear interpolation was applied to achieve the same number of values collected by all sensors (i.e., one value per hour). As an example, if a given sensor recorded 21.57 °C at 02:24 and the next reading was 21.86 °C at 03:37, the temperature estimated by linear interpolation at 03:00 would be 21.713 °C. The calculation is as follows: 21.713 = 21.57 + (21.86 − 21.57) × 36/73, being 36 the timespan in minutes between 2:24 until 3:00, while 73 is the timespan between 2:24 and 3:37. When the timespan of two readings was greater than two hours, the uncertainty derived from linear interpolation seems excessive, and such estimation method was not applied, resulting in one or more missing values.

On the other hand, it turned out that some consecutive data recordings were also lacking in particular nodes due to problems of wireless communication with the gateway, particularly for the most distant nodes. The structure of missing values was checked for all nodes prior to the statistical analysis.

Considering that the monitored period lasted 212 days, which implies 212 × 24 = 5088 h, the application of this method led to a matrix with 5088 rows by 26 columns (nodes). In the context of industrial statistical process control, when a certain parameter is recorded over time, the resulting time series is generally called a trajectory because data usually follow a certain pattern. Hence, in the present work, the sequence of recordings obtained from each node will be referred to hereafter as *trajectory*.

### 2.5. Daily Fluctuations of Temperature

The daily range was calculated as maximum temperature of the day minus minimum. Next, for the time series of daily ranges from each node, a chart of cumulated sums (CUSUM) was obtained using an Excel spreadsheet (Microsoft) by summing, day after day, the mean-centered recorded values. This chart is useful to identify changes of trends along the time. The effect of day of the week was also studied by means of one-way ANOVA using the software Statgraphics 5.1.

### 2.6. Mean Daily Values

The median temperature was also computed per day, which leads to a matrix comprised of 212 observations (days) by 26 variables (sensors). Based on the methodology applied in previous studies aimed at characterizing differences among thermic time series in the context of preservation of cultural heritage [34], data were row centered by subtracting each value in the matrix minus the mean temperature recorded each day (i.e., the row mean). Next, principal component analysis (PCA) was applied by means of the software SIMCA-P 10.0 (Umetrics). 

In order to better highlight the dissimilarities between sensor nodes, PCA was also applied for each of the different stages identified in the period under study. The first principal component (PC1) and the second one (PC2) provide information about the main correlation structures in the matrix. Each component can be interpreted as a linear combination of the original variables (nodes). The contributions or loadings of variables in the formation of PC1 and PC2 will be referred to hereafter as *p[1]* and *p[2]* loadings, respectively. The loading scatter plots corresponding to PC1 and PC2 were obtained for each stage and compared in order to discuss changes in the correlation structure of variables. Based on these plots, a few groups of nodes were identified with a similar pattern in the recorded temperatures, i.e., sensors whose trajectory has an analogous shape and also a similar mean value. Next, data from nodes with a comparable pattern were averaged in order to simplify the number of trajectories shown graphically. The resulting plots allow an easier identification of similarities and dissimilarities among trajectories, which were discussed according to the node position in the church. One plot of this type was obtained for each stage identified along the 7 months of the experimental study.

## 3. Results and Discussion

The structure of missing data is first discussed, followed by the results regarding daily fluctuations of temperature and differences between nodes regarding the trajectories of daily median values.

### 3.1. Discussion of Missing Values

Due to a technical problem with the GSM Internet connection, the gateway was not able to send data wirelessly to the cloud and, after storing them for some time, they were lost. Because of this, data are unavailable on 10 August from 8:00 a.m. until 5:00 p.m. The system also failed from 19 August at 5:00 a.m. until 23 August at 10:00 a.m.

The percentage of data available (i.e., by discounting the number of missing values), which is denoted as data extraction rate (DER), was calculated for each sensor. It was found that four nodes yielded an excessive quantity of missing values:

- **Node OUT**: It disappeared on 1 September. The values are available for just 32 days, but they were taken into consideration because this is the only node inside the narthex, where the microclimate is different.
- **Node SAC**: There is only 23.7% of available data due to problems of communication with the sink gateway. The thick walls separating the central nave with respect to the corridor where this node was placed led to problems of signal propagation, which justifies the higher amount of missing data.
- **Node CH3**: The amount of data collected was much lower for CH3 (54.4%) compared with CH1 (78.5%) and CH2 (75.5%). These three nodes were placed inside the Communion Chapel with thick walls and a metal-cladding wooden door that limited signal propagation and therefore were more affected by the specific positioning. Fortunately, PCA can manage such percentages of missing values.
- **Node LO2**: A total 58.6% of available data. The reasons are uncertain because it is relatively close to the gateway, just 12.5 m away. It was found that the signal level of this specific node was very weak, indicating a flaw in the electronics.

Other authors [43] have reported similar results using wireless sensors in cultural heritage because the data sent by some nodes to the gateway reached 100% while this percentage was lower in others (75–93%). The experience with this deployment and the evolution of radio technology has allowed us to redesign the node for better behavior in historic buildings and lower communication loss due to collisions [62], e.g., our deployment in Alava’s Arms Museum achieves DER higher than 95%.

### 3.2. Daily Fluctuations of Temperature

Starting from the matrix containing 26 variables (nodes) by 5088 rows (hours), the subsequent step was to correct the bias by subtracting every value minus the sensor bias indicated in Table 1. Next, for each node, two parameters were computed: daily temperature range and daily median, resulting one time series of ranges and another of medians. Table 3 shows the average daily range (ADR) for the whole period of 212 days, as well as the average daily median (ADM) computed for days 1–130 (until 8 December). On that date, the heating system of the Communion Chapel was turned on, which introduces a significant distortion in the mean temperature. Nodes OUT and SAC were not included in Table 2 given their high number of missing values. It can be observed that the variability of ADM is small: the lowest value (24.48 °C) corresponds to RE2 and the highest (25.21 °C) to EN2; the difference between them is 0.73 °C.

#### 3.2.1. Identification of Shifts in the Time Series of Daily Ranges

**Figure 6:**
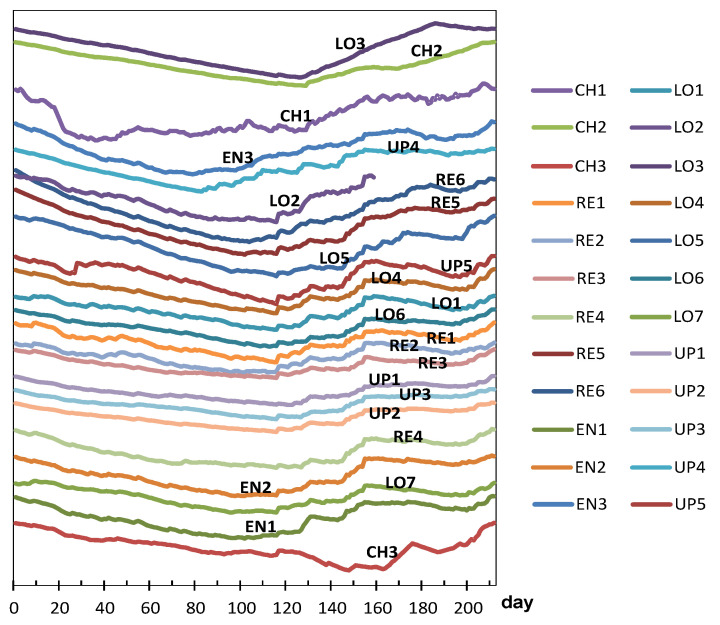
CUSUM charts obtained with the time series of daily ranges of temperature. Charts were conveniently scaled and moved up or down so that those with a similar shape appear close to each other. Nodes OUT and SAC were disregarded. The origin of coordinate, i.e., day 0, corresponds to 0:00 h of 1 August 2017.

For each node, the time series of daily ranges was depicted graphically in order to check if any shift could be identified in the mean or variance at any time point. For this purpose, CUSUM charts were obtained for the 24 mean-centered time series of daily ranges (i.e., after subtracting the average value of each series). These charts are shown in Figure 6 after being conveniently scaled and moved up or down to facilitate their comparison, so that those with a similar pattern were positioned together.

For most nodes, the chart has a shape resembling the letter “V”: the CUSUM sequence firstly decreases, it reaches a relative minimum, and then an increasing pattern is observed. This result indicates a trend change or shift at a particular date. In most cases, such shift occurred at about day 115 (23 November 2017). It is uncertain if any occasional event occurred on this date or if it just reflects seasonal weather changes. Interestingly, nodes with a shift on a different date correspond to those with the highest ADR (Table 3): CH1, UP4, LO3, CH2, and EN3. A more detailed study is required for the time series of daily ranges corresponding to these nodes in order to investigate the possible reasons for the larger temperature ranges.

#### 3.2.2. Nodes with Highest Average Daily Ranges (ADR) of Temperature

**Figure 7:**
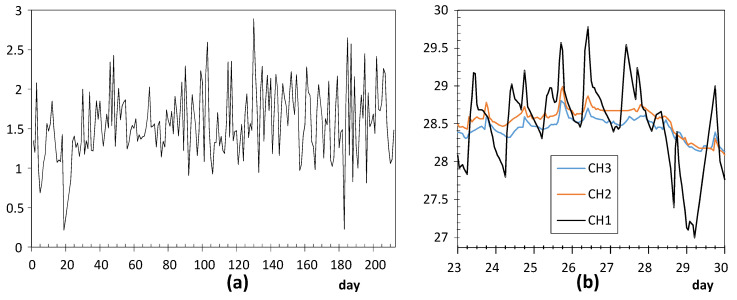
Node CH1: (**a**) time series of daily ranges of temperature (°C) for the whole period of 212 days; (**b**) time series of hourly values of temperature (°C) for the three nodes at the chapel, from day 23 (24 August 2017) until day 29.

**Node CH1**: The time series of daily ranges (Figure 7a) reveals that the mean and variability is approximately maintained over time, with an average of 1.57 °C (see Table 3). The corresponding CUSUM chart (Figure 6) reveals a certain change of trend around day 20. Prior to this date, the trajectory of CH1 was slightly above the average recorded by the other sensors. As this node was placed on a window ledge, results suggest that this window was probably closed before day 20.

It was installed at the Communion Chapel, dropped on a window ledge. This window is east oriented and receives direct sunshine some hours per day. As a result, the glass is heated and creates a different microclimate surrounding this window, which explains the marked fluctuations registered by CH1. The lowest values of daily ranges are probably due to cloudy weather conditions. This pattern of CH1 was neither observed in CH2 nor CH3 because they were placed on a picture frame, far away from any source of fluctuating heat.

**Figure 8:**
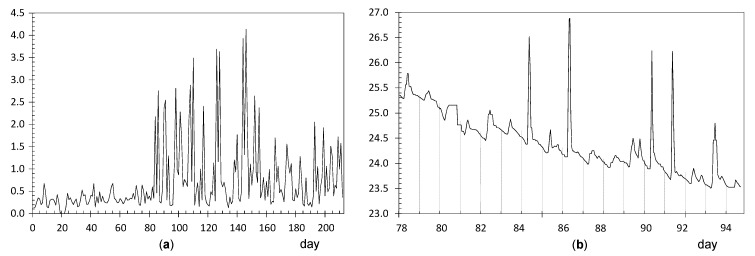
Node UP4: (**a**) time series of daily ranges of temperature (°C) for the whole period of 212 days, (**b**) time series of hourly values of temperature from day 78 (17 October 2017) until day 94.

**Node UP4**: ADR = 0.71 °C. Figure 8a shows a clear shift in the time series of daily ranges around day 84 (23 October). Figure 8b shows the evolution of temperatures from day 78 to 94, i.e., a few days before and after this shift. Daily fluctuations are clearly marked. The first peak occurred on 23 October from 8:00 to 10:00 a.m. As the striking peaks of temperature occurred always at the same time, about 9:00 a.m., they were probably caused by direct sunshine radiation incident on the node through a window located about 6 m away. Similar results have also been reported in related studies [63,64].

**Figure 9:**
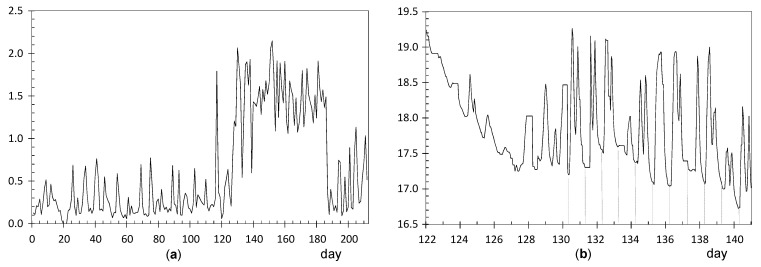
Node LO3: (**a**) time series of daily ranges of temperature (°C) for the 212 days, (**b**) time series of hourly values of temperature (°C) from day 122 (30 November) until day 140.

**Node LO3**: ADR = 0.64 °C. The evolution of daily ranges over time (Figure 9a) reveals a clear shift at about day 130. One-way ANOVA was applied to the period prior to this date, and it was obtained that the effect of day of the week is statistically significant (*p* = 0.0093): temperature fluctuations on Saturdays (average value 0.54 °C) are significantly higher compared with the rest of the days of the week (average of 0.27 °C). The reason might be the presence of people attending the mass service on Saturday afternoon at the central nave, while the liturgical service is carried out during weekdays (Monday to Friday) at the Communion Chapel. The same ANOVA model was repeated for the period after day 130, but it turned out that differences were not statistically significant (*p* = 0.91).

**Figure 10:**
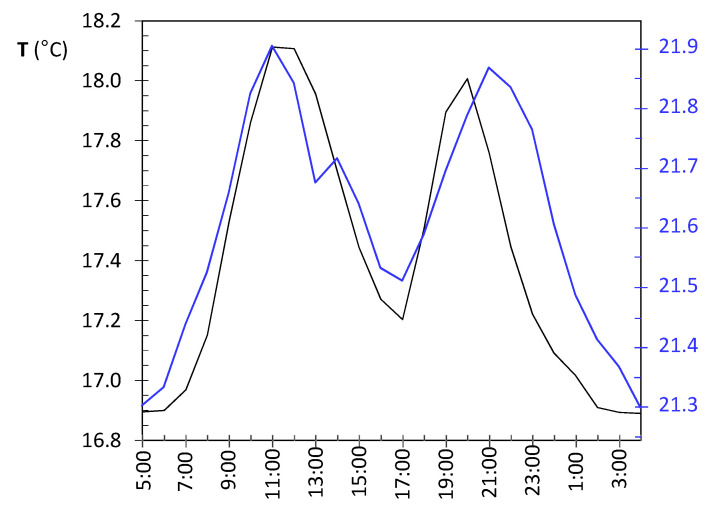
Daily evolution of temperature for node LO3 (black trajectory), averaged from 5 December until 2 February (values in °C indicated on the left scale), and for node CH2 (blue trajectory), averaged from 11 December until 2 January 2018 (values on the right scale).

The shift at day 130 is coincident with the assembly of the nativity scene next to the confessional where node LO3 was dropped on the top. A specific light system was installed for this crèche, so that a halogen lamp remained relatively close to the node, which explains the higher temperature fluctuations. Figure 9b shows the temperature evolution during some days before and after the date when the shift occurred. Daily cycles are clearly observed but, curiously, instead of cycles with a sinusoidal shape which might be expected, two peaks are observed in most days. The period comprised of this particular pattern was between day 127 (5 December) until day 186 (2 February), when the nativity scene was put away. If the daily trajectory of temperature is averaged for this period (Figure 10), the presence of two peaks is noticeably observed. Lamps illuminating the nativity scene were turned on from 8:30 a.m. until 1:00 p.m. (midpoint 10:45 a.m.), which is rather coincident with the first peak that occurred around 11:30 a.m. In the afternoon, the church opens for mass service and the lamps are turned on again from 6:30 p.m. to 8:50 p.m. (midpoint 7:40 p.m.), which would explain the second peak observed around 8:00 p.m. From 1:00 p.m. until 6:30 p.m., the temple remains closed, and the lights are off from 1:00 p.m. until 6:30 p.m., which would justify the temperature drop observed in Figure 10, considering the presence of a halogen lamp at a short distance of node LO3.

**Figure 11:**
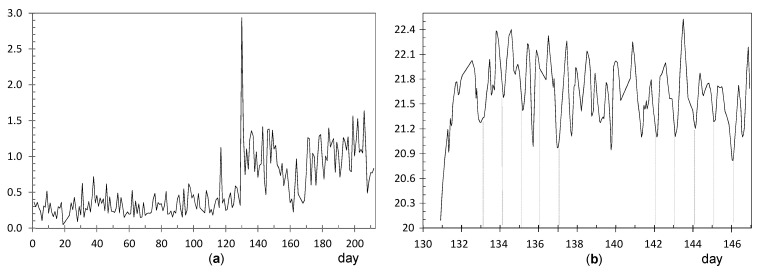
Node CH2: (**a**) time series of daily ranges of temperature (°C) for the 212 days, (**b**) time series of hourly values of temperature from day 130 (8 December) until day 146 (24 December).

**Node CH2**: The evolution of daily fluctuations is shown in Figure 11a, being the average value ADR = 0.58 °C. The highest peak corresponds to day 130 when the heating system at the chapel was started. Afterwards, daily ranges tended to be greater, probably because the heating system did not work uniformly during the day.

The trajectory from day 130 onwards reflects daily oscillations (Figure 11b). The period between days 133 and 155 (11 December until 2 January) was selected based on Figure 11a, considering the decrease in daily fluctuations from days 150 up to 170. For this period, the mean daily trajectory was calculated, which is depicted in Figure 10 in blue. It strongly resembles the daily pattern of LO3 (Figure 10 in black). Thus, when the church was closed from 1:00 p.m. until 6:30 p.m., the temperature decreases about 0.3 °C on average. The main reason seems to be the heating system in the chapel, which is connected from 8:30 to 11:15 a.m. and, then, from 5:00 p.m. to 9:00 p.m. Nevertheless, there might be other factors involved, such as lighting or the opening routines of windows.

**Figure 12:**
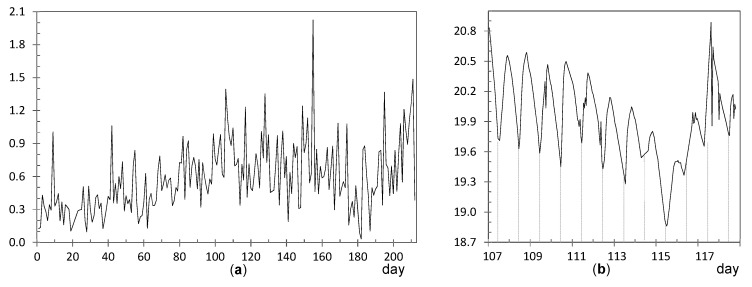
Node EN3: (**a**) time series of daily ranges of temperature (°C) for the 212 days, (**b**) time series of hourly values of temperature from day 107 to 118 (15 to 26 of November).

**Node EN3**: ADR = 0.59 °C. The CUSUM chart (Figure 6) reveals a shift at about day 80 (19 October). Daily ranges after this date tended to be slightly higher (Figure 12a) and, moreover, daily cycles are clearly marked (Figure 12b), reaching a relative minimum around 8:00–10:00 a.m. A different pattern is reflected in Figure 10, which shows a relative maximum at 10:00–11:00 a.m. This node EN3 was located on the top of a door frame at the narthex, facing inside the building. It was observed that direct sunshine radiation impacts this door at a certain timespan in the morning when the main entrance of the church is open, which would explain the higher fluctuations. Given that such radiation varies along the year, it seems that sunshine did not affect the node before day 80. Such higher ranges were neither observed in EN1 nor EN2 because they were located on the narthex roof, which did not receive direct sunshine.

**Figure 13:**
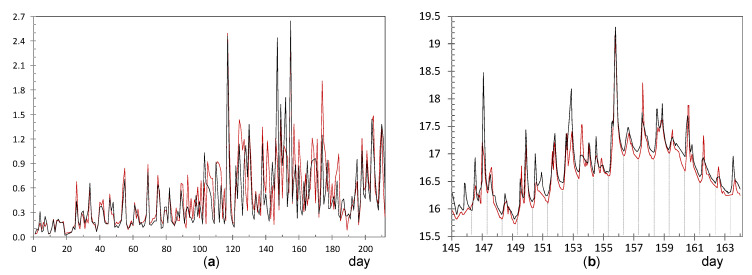
Evolution of temperature (°C) for nodes RE5 (black trajectories) and RE6 (brown): (**a**) time series of daily ranges for the 212 days, (**b**) time series of hourly values from day 145 (23 December) up to day 163 (10 January 2018).

**Nodes RE6 and RE6**: They are located at the upper part of the altarpiece in a symmetrical position, which explains the similar shape of their trajectories (Figure 13a). The higher variability seems to be caused by the presence of nearby halogen lamps, at about 4 m away, which heat the nodes when they are turned on. According to their respective CUSUM charts (Figure 6), the shift occurred in both cases around day 104, on 12 November, about 11 days earlier than most of the rest of nodes. In both nodes, two pronounced peaks are observed at day 117 (25 November) and 155 (2 January), as reflected by Figure 13b. Both correspond to special celebrations so that lamps were turned on for longer, which would explain the higher peaks.

**Figure 14:**
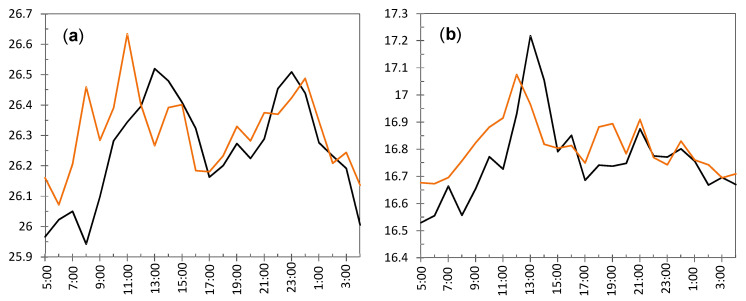
Daily evolution of temperature (°C) for nodes RE5 (brown trajectories) and RE6 (black): (**a**) averaged for days 1 to 104, (**b**) averaged for days 105 to 212.

Figure 13b shows the trajectories for 19 days, which are similar in both cases. Daily fluctuations are clearly marked. In order to further study this pattern, and considering the shift occurred at day 104 (Figure 13a), the daily mean trajectory was computed up to this date (Figure 14a). Certain resemblance appears with respect to Figure 10. Minimum values are reached at night, then temperature increases and reaches a relative maximum at solar midday (from 11:00 a.m. to 2:00 p.m.). Afterwards, the temperature undergoes a relative minimum at about 4:00 p.m. to 6:00 p.m. because the church remains closed and lights are off, and finally the recordings rise again and reach a second maximum. If daily mean trajectories are computed for days 105 to 212 (Figure 14b), it turns out that the temperature drop at nighttime is less pronounced even when the church closes.

#### 3.2.3. Study of Daily Trajectories

Given the particular pattern reflected by Figure 10, a further study was carried out in order to characterize the differences between nodes with respect to their daily mean trajectories. For this purpose, the period from day 82 (21 October) to 129 (7 December 2017) was chosen. The subsequent days were disregarded because temperatures in the chapel are affected by the heating system, and hence their daily pattern cannot be directly compared with the rest. For this period of 48 days, the daily mean trajectory was computed for each node by averaging the values collected at each hour and we then wrote down the time when the minimum temperatures were reached. Moreover, we noted if a drop was observed around 5:00 p.m., such as in the case of Figure 10 and Figure 14a. It was found that nodes RE (Figure 14) and UP4 are the ones with the most different daily trajectory. For the latter, the presence of sudden peaks was caused by sunshine radiation. Regarding the rest of the nodes, the most dissimilar daily trajectory was observed in those located at the entrance, EN1, EN2, and EN3; minimum values are reached at about 9:00 a.m. or 10:00 a.m., and one single relative maximum is achieved at about 7:00 or 8:00 p.m. However, for the rest of the nodes, except RE6 and UP4 as already mentioned, two relative maximums are observed. In most cases, the minimum temperature is reached between 7:00 and 9:00 a.m., while the first maximum occurs between noontime and 1:00 p.m. This first peak is usually higher than the second except in the case of RE2, LO1, LO2, LO4, CH2, and CH3. Curiously, LO1 is located relatively close to the main entrance and its trajectory is somewhat like that of EN1, which is intuitively appealing.

#### 3.2.4. Study of the Effect of Week Day

CUSUM charts of daily ranges reveal a shift that occurred in most nodes around day 115. Based on this information, one-way ANOVA was performed for each node considering one factor with seven variants (days of the week). The analysis was carried out firstly for the period of days 1–115, and it was found that the effect of day of the week was statistically significant (considering ***α*** = 5%) in 15 nodes: EN1, EN2, LO1, LO2, LO3, LO4, LO6, UP1, UP2, UP3, RE2, RE3, RE4, RE5, and RE6. In all of them, the means plot with LSD intervals revealed that higher temperatures were recorded on average on Saturdays and Sundays. However, the ANOVA was repeated for the interval of days 116–212, and the effect of the day of the week did not appear as statistically significant (*p* > 0.1).

By computing mean values, the daily range from Monday to Friday is about 0.22 °C, on average, but it becomes nearly double on Saturdays, 0.42 °C, and slightly lower on Sundays, 0.36 °C. Such higher fluctuations can be explained by the fact that mass services on weekends are celebrated at the high altar of the central nave, while the Communion Chapel is used from Monday to Friday given the lesser attendance and because this chapel is more comfortable in winter given the heating system. On weekends, the considerable amount of people at the central nave and the enriched lighting used for the celebrations would explain the statistically significant increase in daily ranges on Saturdays and Sundays, which is about 0.2 °C on average.

### 3.3. Differences between Nodes Regarding the Trajectories of Daily Median Temperature

**Figure 15:**
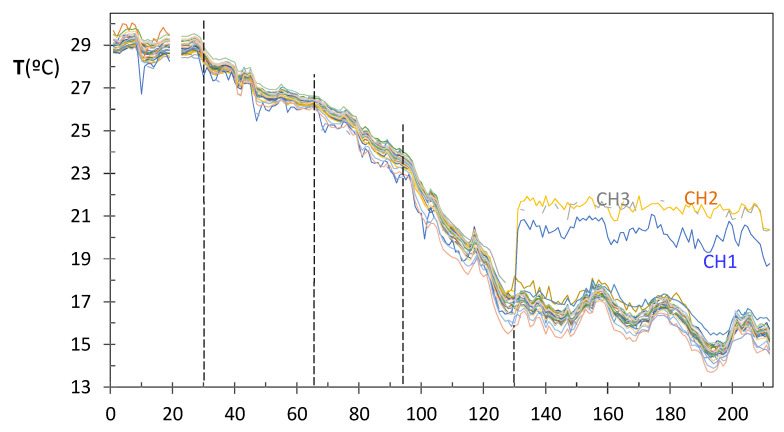
Time series of daily median temperatures for the whole period of 212 days. Vertical dashed lines indicate a change of trend or slope, identified at days 30, 66, 94, and 130.

One matrix was assembled consisting of 212 observations (days) by 26 columns (nodes), which contains the median temperature recorded daily. The monitoring period started in summer (1 August) and ended in winter. Generally speaking, all trajectories follow a monotonously decreasing trend (Figure 15) and are mainly parallel. For the three nodes at the Communion Chapel, a sudden shift is observed on 8 December at 2:00 p.m., feast of the Immaculate Conception. After this date, the mean temperature was 20.2, 21.4, and 21.3 °C for CH1, CH2, and CH3, respectively, because the radiant floor heating system was turned on. The set point was fixed at about 21 °C, which agrees with the recommendation of the Spanish Government’s Institute for the Diversification and Saving of Energy [65] to set the thermostat around 20–21 °C for homes during the day.

Although the underfloor heating system keeps an adequate average temperature for artworks at 20–22 °C, sudden changes in temperatures should always be avoided. In this case, the temperature increased 4 °C in one day, which seems excessive. It should be advisable to activate the heating system when the average temperature in this chapel drops below 20 °C, which corresponds to day 100. This is also in accordance with the EN 16,883:2017 standard [11] and the concept of a green museum [15,16] since energy consumption is minimal when the temperature difference is small.

Apart from the shift clearly observed in Figure 15, PCA allows the identification of other changes in the correlation structure of variables which are not so apparent. The matrix of daily medians was row centered by subtracting each value minus the row average. The mean trajectory of all nodes is the main source of data variability, which is removed using this procedure. Therefore, the subsequent PCA model will characterize the dissimilarities between trajectories and will identify those ones slightly different from the rest. Time-frequency analysis is another well-researched domain for the classification of sensor data [45,46,47]. Another approach is to extract parameters from the time series and apply classification methods [6,66]. The comparison of these different approaches is out of the scope of the present work.

PCA results revealed an abnormal behavior for CH nodes, which is apparent in Figure 15, from day 130 onwards. Thus, it seems reasonable to split the monitored period in at least two stages: days 1–130 and 131–212.

#### 3.3.1. PCA for the Period of Days 1–130

**Figure 16:**
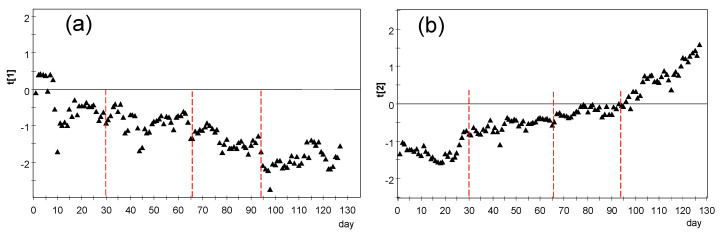
Results from the PCA applied to the matrix of daily medians for the period of 130 days: (**a**) evolution of scores corresponding to PC1, (**b**) evolution of scores corresponding to PC2.

After selecting data from the first 130 days, PCA was repeated considering all 26 nodes. The following values were obtained regarding the percentage of data variability explained by each component: PC1, 62.9%; PC2, 25.0%; PC3, 4.4%; and PC4, 3.5%. One criterion commonly considered is to focus the attention on those PCs with an eigenvalue λ > 1, which is the case for PC1 (16.4), PC2 (6.5), and PC3 (1.1), but neither for PC4 (0.9) nor further components. Thus, it can be concluded that PC1 and PC2, which account for 87.9% of the data variability, basically synthetize the most relevant information. Figure 16 shows the evolution versus time of scores corresponding to PC1 and PC2, which will be denoted hereafter as *t[1]* and *t[2]* scores, respectively. A marked evolution is observed in both cases. Considering that the mean trajectory of all sensors was removed prior to applying PCA, this result indicates that the correlation structure is changing over time.

In order to better characterize this evolution of microclimate conditions, four sub-periods were considered based on Figure 15 and Figure 16: days 1–30, 31–66, 67–94, and 95–130. According to Figure 15, the mean temperature is basically constant up to day 30 (summer); next, there is a progressive temperature decrease that becomes more pronounced from day 66 onwards and on day 94. Thus, an individual PCA model was performed for each stage, as discussed next.

#### 3.3.2. PCA Results for the Period of Days 1–30

**Figure 17:**
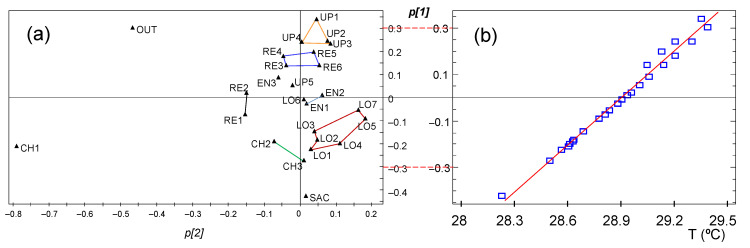
PCA applied to daily medians for the period of days 1–30: (**a**) loading plot for PC1 and PC2 (*p[1]* vs. *p[2]* loadings), (**b**) scatterplot of *p[1]* vs. mean temperature in that period. The fitted regression line is indicated in red.

In this case, PC1 and PC2 explain 81.4 and 13.4%, respectively, of the data variability, while PC3 accounts just for 1.9% (λ = 0.49). It was checked that *p[1]* loadings are strongly correlated with the mean temperature collected by nodes (*r* = 0.993), as shown in Figure 17b. SAC is the node with the lowest *p[1]* value, which implies the lowermost mean temperature, probably because the corridor leading to the sacristy is isolated from the central nave, and none of the walls receive direct sunshine. Regarding *p[2]* loadings, CH1 and OUT appear in Figure 17a as outliers. OUT was installed inside the narthex and the environment here is intermediate of air conditions inside and outside the building. This is the node with the second highest mean values, which is consistent with the hot temperatures reached in the city of Valencia in summer.

**Figure 18:**
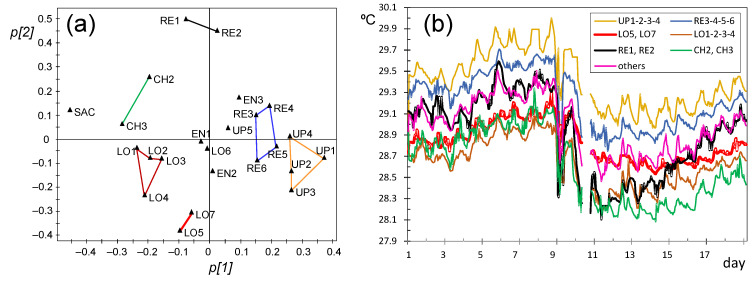
PCA of daily medians for the period of days 1–30 after discarding nodes CH1 and OUT: (**a**) loading plot for PC1 and PC2 (*p[2]* vs. *p[1]* loadings), (**b**) time series of hourly values from day 1 to 18 (1–18 of August 2017). Nodes appearing close to each other in (**a**) were joined together and their trajectories were averaged in (**b**). “Others” accounts for EN1, EN2, EN3, UP5, and LO6.

The fact that PC2 is strongly influenced by OUT and CH1 indicates that their trajectories are somewhat different, which is reasonable given their unique position: the former was placed inside the narthex and the latter next to a window partly open. If a new PCA is fitted after discarding both nodes, PC1 and PC2 explain 92.1 and 3.5%, respectively. This result implies that trajectories will be rather parallel. The loading plot for PC1 and PC2 is depicted in Figure 18a. As in the case of Figure 17b, a tight correlation exists between *p[1]* and the mean temperature collected during the first 30 days. Thus, the plot reveals that the highest average values were recorded by nodes at the upper position (UP), while the opposite applies to SAC. The reason seems to be that the ceiling of the church is hotter than the walls due to the high outer temperatures in summer, and it heats the mass of air at the top of the building.

Regarding PC2, it accounts for the different shape of trajectories and highlights those nodes with a trend that departs from the common pattern. RE1 and RE2 appear in the loading plot oppositely located with respect to LO5 and LO7, which implies that their trajectories will be the most dissimilar. In order to visualize such differences, the following procedure was applied. Firstly, nodes installed near to each other in the church and appearing close together in the loading plot were joined as a cluster (Figure 18a) because their collected values will be similar. Next, average trajectories were computed for each cluster, which are displayed in Figure 18b. A group of nodes on a central position in the loading plot were grouped as “others”.

A clear shift is observed in Figure 18b around day 9, on 9 August. Trajectories are rather parallel before this date and daily cycles are coincident. However, after day 9, the parallel trend is no longer maintained because LO5 and LO7 (red trajectory) follow a different pattern, with a lower slope compared with most of other nodes. As a reminder, LO5 and LO7 were located symmetrically on the marble lintel above a big door, while LO1, LO2, LO3, and LO6 were dropped on the top of a wooden confessional. Thus, the different position of LO5 and LO7 would justify their dissimilarity with respect to the rest of LO nodes. Conversely, the average trajectory of RE1 and RE2 (in black) is steeper, but not for the other nodes at the altarpiece (blue trajectory: RE3-4-5-6). As such, the opposed pattern of LO5-LO7 compared with RE1-RE2 justifies their opposite position in Figure 18a. Nodes RE1 and RE2 are located on the base of a niche holding a statue, and it turns out that both underwent a more abrupt temperature drop after day 9, but their trajectories tended to equilibrate and recover their relative position as in the first days.

In summary, the interpretation derived from PCA analysis is similar to previous work [35], concluding that PC1 could be interpreted as the yearly average, while PC2 provided basic information about deviations with respect to the general trend.

#### 3.3.3. PCA Results for the Period of Days 31–94

**Figure 19:**
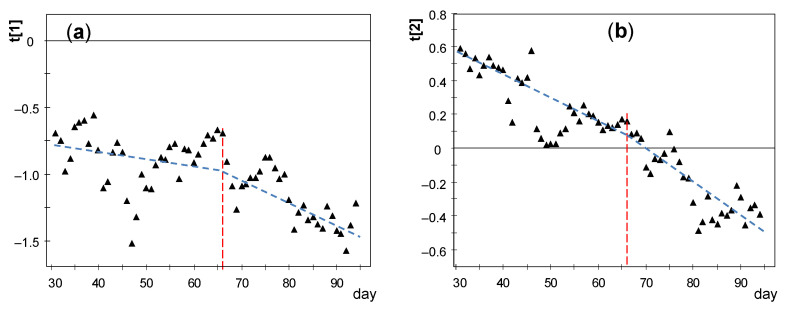
PCA of daily medians for the period of days 31–94 after discarding nodes CH1 and OUT: (**a**) evolution of scores corresponding to PC1, (**b**) evolution of scores corresponding to PC2.

Node OUT presents 97% of missing values in this stage and it was discarded for the rest of the study. It was checked that CH1 has a strong influence on the relevant components as in the previous stage (Figure 17a) and it was also disregarded. By performing a new PCA, it turns out that PC1 and PC2 account for 84.2 and 8.5% of the data variability, respectively, being λ_PC2_ = 2.03. The evolution of *t[1]* and *t[2]* scores versus time reveals a linear trend (Figure 19), which implies that the correlation structure was not stable during this stage. In order to better characterize this evolution, the period was split in two parts with a similar duration: days 31–66 and 67–94. The cut-off was chosen at day 66 because a certain change of trend is observed on this date in Figure 15 and Figure 19.

#### 3.3.4. PCA Results for the Period of Days 31–66

**Figure 20:**
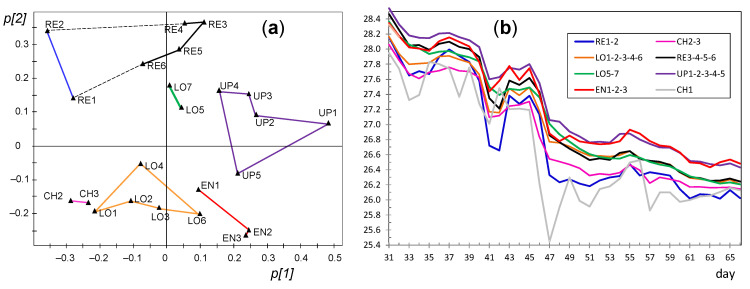
PCA of daily medians for the period of days 31–66 after discarding nodes CH1, OUT, and SAC: (**a**) loading plot for PC1 and PC2 (*p[2]* vs. *p[1]* loadings), (**b**) time series of daily median temperatures from day 31–66. Trajectories were averaged according to clusters indicated in (**a**).

In this stage, SAC has too many missing values and it was removed. As already discussed, CH1 has a strong influence on the relevant components, and it was also disregarded. A new PCA was carried out next for days 31–66, and it turned out that PC1 explains 84.3% of the data variability while PC2 only 5.7% (λ = 1.3). The evolution of *t[1]* and *t[2]* scores does not reflect any trend (figure not shown). If the resulting loading plot (Figure 20a) is compared with Figure 18a, some differences are apparent regarding the position of certain nodes, particularly those with highest influence on PC2. All RE nodes located at the altarpiece appear in the plot opposite to those at the entrance (EN1, EN2, EN3) and chapel (CH2 and CH3), which implies differences in their respective trajectories. Aimed at visualizing such dissimilarities, nodes close to each other in Figure 20a were clustered and, then, their trajectories were averaged.

Taking into account that *p[1]* loadings are strongly correlated with the mean, a lower temperature was observed for RE1 and RE2 compared with the rest of nodes at the retable. The reason could be that both nodes are located close to a thick wall supporting the altarpiece, so that their recordings seem to be influenced by the temperature of that wall. The changes in the correlation structure of variables between days 1–30 versus 31–66 can be discussed by comparing Figure 18a and Figure 20a. The main difference corresponds to nodes at the altarpiece, which now appear closer to each other compared with the first stage. Generally speaking, UP nodes tended to record higher temperatures than those at lower positions, coded as LO, probably because hot air tends to rise. A careful inspection of Figure 20b reveals that RE nodes underwent quicker temperature drops (e.g., at days 41 and 47) compared with the rest. Conversely, LO5 and LO7 had a smoother trajectory, i.e., they were less affected by temperature drops induced by outer weather conditions. This different pattern between LO5 and LO7 with respect to RE nodes is not easily explained given their proximity (Figure 5).

#### 3.3.5. PCA Results for the Period of Days 67–94

**Figure 21:**
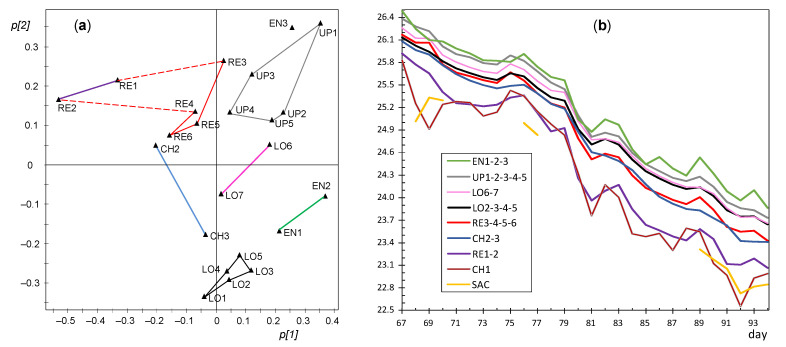
PCA of daily medians for the period of days 67–94 after discarding nodes CH1, OUT, and SAC: (**a**) loading plot for PC1 and PC2 (*p[2]* vs. *p[1]* loadings), (**b**) time series of daily median temperatures from day 67–94. Trajectories were averaged according to clusters indicated in (**a**).

In this case, PC2 is strongly influenced by SAC and CH1. Hence, a new PCA was applied after removing both nodes as well as two abnormal (Figure 21a). Results reveal a strong correlation because PC1 explains 93% of the data variability, while PC2 only 2.5% (λ = 0.95). These values suggest that trajectories are rather parallel, as reflected by Figure 21b. Nevertheless, given that most LO nodes exert an influence on PC2 and appear in the plot opposite to UP nodes, some differences are expected in their trajectories. Around day 67, the mean temperature of UP nodes was 26.4 °C, while it was 26.1 °C for LO2-3-4-5, i.e., a difference of 0.3 °C. However, their decrease was slightly unalike, so that at day 94 the difference of mean temperature between them was 0.1 °C.

The lowest temperatures were recorded by SAC, CH1, RE2, and RE1. The corridor leading to the sacristy is ventilated through a window that allows entrance of outer air, which would justify the lower temperature recorded by SAC compared with the central nave. Similarly, CH1 is located on a window ledge, and the exchange of outside air through the partly open window would justify the cooler environment. The lower temperatures of RE1 and RE2, as discussed above, seem to be caused by their proximity to a thick wall. In the present research, it is not possible to study whether the type of material closest to the node had any effect on the recordings. Actually, some nodes were dropped on a wooden surface, others were fastened to a banister, but few nodes were located on thick walls. Thus, the effect of the different thermal diffusivity or conductivity of the materials surrounding the nodes is uncertain. This issue should be taken into consideration in further studies.

#### 3.3.6. PCA for the Period of Days 95–130

**Figure 22:**
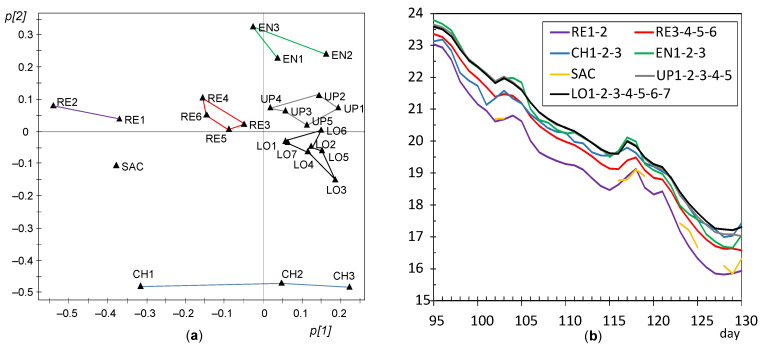
PCA of daily medians for the period of days 95–130 after discarding node OUT: (**a**) loading plot for PC1 and PC2 (*p[2]* vs. *p[1]* loadings), (**b**): time series of daily median temperatures from day 95–130. Trajectories were averaged according to clusters indicated in (**a**).

Another PCA model was fitted for this period considering all sensors except OUT. PC1 and PC2 account for 86.5 and 7.1%, respectively, of the data variability, being λ_PC2_ = 1.8. Nodes tend to appear in the loading plot close to others with a similar position in the church (Figure 22a), so that it is possible to establish five well-defined clusters: RE, EN, UP, LO, and CH. As in the previous stage, it can be deduced that the lowest temperatures were recorded by RE1 and RE2. PC2 is strongly influenced by CH nodes, which appear in the loading plot opposite to EN nodes. In order to discuss their dissimilarities, Figure 22b shows the mean trajectories for clusters established in the loading plot. Although all the time series are quite parallel, it can be observed that EN nodes (green trajectory) underwent a temperature drop slightly faster than most of the rest, while the opposite occurred with CH nodes (in blue), so that their trajectories cross around day 119. The environment in the chapel was somewhat buffered (i.e., slightly more stable) than in the central nave given its smaller size, so that in winter it became less affected by cool temperatures from outside. Conversely, EN nodes were probably more influenced by the entrance of outer air which was cooler than the environment within the central nave, which would explain why the temperature decrease was slightly faster in the case of EN nodes.

#### 3.3.7. PCA Results for the Period of Days 131–212

A marked shift is observed in Figure 15 at day 130 for the three nodes at the chapel when the heating system was turned on. Hence, they were excluded for a subsequent PCA model in order to better characterize the dissimilarities of the remaining trajectories. As this shift strongly affects the average temperature recorded by the set of nodes, the row centering procedure was reapplied after disregarding OUT and CH nodes, prior to applying PCA. By using this procedure, it turns out that PC1 and PC2 explain 85.6 and 4.7%, respectively, of the data variability, being λ_PC2_ = 1.04. The evolution of *t[1]* and *t[2]* scores versus time (figures not shown) does not reveal any clear trend, which suggests that the correlation among variables was more or less maintained along this period.

**Figure 23:**
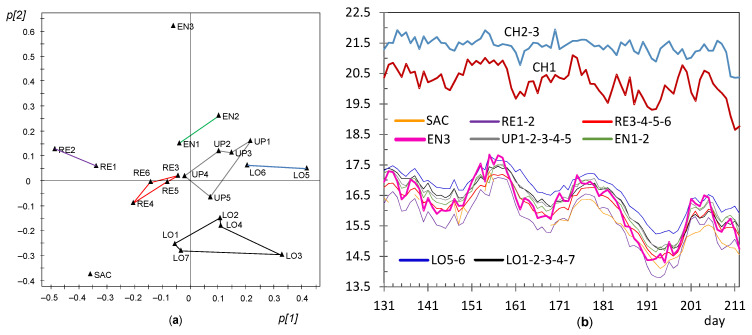
PCA of daily medians for the period of days 131–212 after discarding nodes OUT, CH1, CH2, and CH3: (**a**) loading plot for PC1 and PC2, (**b**) time series of daily median temperatures from day 131–212. Trajectories were averaged according to clusters indicated in (**a**).

The mean temperature of CH1 was about 1 °C below that of CH2 and CH3, probably because the former was located near a window that remained partly open, allowing certain exchange of cool air from outside. It can be observed that PC2 is strongly influenced by EN3, which will correspond to the most dissimilar trajectory. This issue is confirmed in Figure 23b: most trajectories are nearly parallel, but EN3 is the one that departs from the common pattern because temperature cycles are slightly more pronounced, i.e., with greater amplitude. This result can be explained according to the position of EN3, on the top of a door frame at the narthex facing inside the building. Sunshine radiation falls upon the surface of this door when the main entrance is open, at certain timespans of the day. It seems that this radiation lasted for longer at this time of the year. EN1 and EN2 also appear with positive *p[2]* loadings, which is intuitively appealing because they were located on the narthex roof and hence there was also a slight influence of the microclimate inside this wooden structure. Conversely, SAC and all LO nodes appear with negative *p[2]* loadings, which indicates the opposite performance, i.e., their cycles were more buffered, with a lower amplitude, which reveals a microclimate more stable.

By comparing Figure 22a (days 95–130) and Figure 23a (days 131–212), it can be deduced that LO5 underwent a relative increase in temperature between the two stages, being the node with highest average temperature outside the chapel. LO5 was located above the chapel door, so that the warmer air from within partly escaped when the door was open, affecting the environment surrounding this node. Moreover, LO3 also registered a relative increase in temperature compared with the previous period, probably because the sensor was exposed to heat radiation by a nearby lamp installed for lighting the nativity scene placed on day 130 next to the confessional supporting this node.

If the loading plots of the different stages are compared (Figure 17a, Figure 18a, Figure 19a, Figure 20a, Figure 21a, Figure 22a and Figure 23a), it can be noticed that the relative position of RE and UP nodes is not maintained. In summer, the temperature at the top of the central nave (UP) tended to be higher than at lower levels (LO nodes), probably because the roof reached relatively high temperatures in this season. However, such differences decrease progressively and the two clusters (i.e., UP vs. LO nodes) tend to converge in winter. On the other hand, as already discussed, it is striking that nodes RE1 and RE2 at the bottom of the altarpiece were the ones which recorded the lowest temperature on average in summer, like SAC (Figure 23a).

In order to further investigate the differences between summer and winter, the period of days 16–29 was selected as well as another set of 11 days in autumn before turning on the heating system at the chapel: days 119 to 129. If the mean temperature of the period is computed for each node, it turns out that the correlation between both stages is not statistically significant (*p* = 0.8). This result implies a changing microclimate, not only because there is a progressive temperature decrease from summer to winter but also because the correlation structure among nodes is not maintained.

## 4. Conclusions

This work describes the design and implementation of an IoT wireless monitoring system suitable for the environmental monitoring needs of historic buildings. The data collected has been statistically evaluated for two purposes: (i) to characterize the differences between points of daily temperature fluctuations and mean values aimed at better understanding the factors explaining such differences and (ii) to practically evaluate the suitability of this type of monitoring system for the characteristics of the data analysis intended.

It might be argued that the present work is presented as a stand-alone methodology, not referring to other methods for comparison. Our research group is currently working on alternative statistical approaches for the classification of sensor nodes in the context of microclimate monitoring applied to cultural heritage [66]. The main purpose of the present work was to discuss the main sources of temperature variability in a particular church, and the methodology used here turned out to be very powerful for that purpose. A detailed comparison with alternative statistical methods is out of the scope of the present work because it would lead to a very long paper.

The type of statistical analysis performed required the bias correction of the sensors, being extremely important to perform this task before the deployment of the nodes. By working with bias-corrected values, the comparison of results from the statistical analyses carried out has derived very useful information about the criteria to decide the best position for thermic sensors. For example, it seems convenient to locate some nodes close to the main sources of air exchange with the outer environment. In ancient churches, ventilation is usually achieved through open windows and doors, particularly the main entrance. It is important to properly choose the position of nodes in such cases, but certain considerations must be considered. For example, east-oriented windows can receive direct sunshine radiation, which would produce marked temperature fluctuations in nearby sensors. It is recommended to locate nodes close to the points of airflow exchange, such as doors or windows, but the effect of radiation from heated glasses should be avoided as well as the impact of direct sunshine on nodes. Results reported here have also revealed that nodes placed near halogen lamps may also record higher temperature fluctuations. Thus, sensors should not be positioned close to those lamps unless there is a particular interest.

Height is another factor for deciding the best location of sensors. In the present work, the position of some nodes at the top (UP) and others at a lower position (LO) has resulted as appropriate to assess the different temperatures according to height. However, it would be useful to install a few nodes at an intermediate position between the floor level and roof, to estimate temperature gradients more accurately.

Further criteria for an appropriate position of nodes include the following. To check the accuracy of the monitoring system, it seems convenient to put a few nodes at symmetric positions where similar microclimate conditions are expected. Moreover, it is important to measure the air temperatures outside the building by using one or two nodes, if possible. As heritage buildings are usually comprised of several rooms, it is obviously of interest to install nodes in each one for assessing the different environments. Nevertheless, this goal is not always possible to achieve using the current wireless nodes due to problems of communication with the sink gateway. As a rule of thumb, the best locations are those where convection air movements are not hampered. Finally, based on the results obtained for RE1 and RE2, it seems that additional nodes should have been placed close to the thick walls. Assessing the different temperature over time between indoor conditions and walls is of interest to better understand the dissimilarities among nodes. Based on the results, the recommendation for a monitoring system in ancient churches or similar heritage buildings with low ventilation rate and without heating sources would be to use a minimum of about 5 sensor nodes and a maximum of 15. Using more than 15 seems redundant, except in particular cases. Moreover, less than five nodes might be insufficient in big buildings if there is an interest to study the differences according to height and the effect of the ventilation.

Regarding the performance of the wireless monitoring system, it allowed real-time data collection for seven months without the need for manual downloading, but some reliability issues have compelled us to review certain aspects. This experience has allowed us to evolve towards systems that better address the issues of historic buildings with greater distances covered, better through-wall performance, a 10-year maintenance-free battery life, and fixed environmental sampling time patterns to facilitate subsequent statistical analysis [67].

An environmental study using statistical techniques, such as the one carried out in this work, is essential before undertaking actions to correct the indoor climate, whether they are simple actions, such as opening and closing doors and windows, or more far-reaching, such as changes to lighting or heating systems. The use of IoT technology is providing essential opportunities for quick and easy deployment of environmental monitoring systems to gather information for such analysis. This is especially interesting in hard-to-reach places, such as high areas of historic buildings where maintenance is not easy to perform or in remote locations where it is difficult to ensure continuous supervision by an operator.

## Figures and Tables

**Table 1:** Temperature bias (°C) for each node, derived from the calibration experiment.

Node	Bias	Node	Bias	Node	Bias	Node	Bias
CH1	−0.075	LO1	−0.003	RE1	−0.036	UP1	−0.277
CH2	0.012	LO2	0.069	RE2	−0.046	UP2	−0.098
CH3	0.083	LO3	−0.088	RE3	−0.249	UP3	0.276
EN1	−0.280	LO4	0.077	RE4	0.000	UP4	0.335
EN2	0.097	LO5	0.009	RE5	0.150	UP5	0.175
EN3	0.160	LO6	−0.019	RE6	0.189		
OUT	−0.028	LO7	−0.089	SAC	−0.115		

**Table 2:** Distance of the nodes (height, in m) from the floor level of the central nave of the church.

Node	Height	Node	Height
CH1	4.2	RE1, RE2	4.0
CH2, CH3	3.7	RE3, RE4	8.6
UP1, UP2, UP3, UP4	12.1	RE5, RE6	12.2
UP5	5.0	EN1, EN2	2.5
LO1, LO2, LO3, LO6	2.0	EN3	4.3
LO4	3.9	SAC	3.5
LO5, LO7	5.0	OUT	2.0

**Table 3:** Summary parameters of temperature for 24 nodes: average of daily ranges (ADR, °C) computed from day 1 to 212 and average of daily median (ADM, °C) up to day 130.

Node	ADR	ADM	Node	ADR	ADM	Node	ADR	ADM
CH1	1.57	24.49	LO3	0.64	25.04	RE4	0.40	24.94
CH2	0.58	24.82	LO4	0.28	24.95	RE5	0.48	24.64
CH3	0.34	25.06	LO5	0.22	25.05	RE6	0.53	24.58
EN1	0.37	25.05	LO6	0.47	25.11	UP1	0.43	25.06
EN2	0.40	25.21	LO7	0.20	24.99	UP2	0.38	24.96
EN3	0.59	25.10	RE1	0.35	24.60	UP3	0.39	24.87
LO1	0.33	24.84	RE2	0.34	24.48	UP4	0.71	25.11
LO2	0.37	24.96	RE3	0.33	25.05	UP5	0.40	25.14

## Data Availability

Data available in Perles, A.; Zarzo, M.; Mercado, R.; García-Diego, F.-J. (2021). Temperature measurements at Saint Thomas and Saint Philip Neri church. Zenodo 2021. https://doi.org/10.5281/zenodo.5651453 (accessed 17 November 2021).
